# Outcome of the “Manchester Groin Repair” (Laparoscopic Totally Extraperitoneal Approach With Fibrin Sealant Mesh Fixation) in 434 Consecutive Inguinal Hernia Repairs

**DOI:** 10.3389/fsurg.2018.00053

**Published:** 2018-09-18

**Authors:** J. James Pilkington, M. Rami Obeidallah, M. Saad Zahid, Panagiotis Stathakis, Ajith K. Siriwardena, Saurabh Jamdar, Aali J. Sheen

**Affiliations:** ^1^Department of General Surgery, Manchester Royal Infirmary, Manchester University Foundation Trust, Manchester, United Kingdom; ^2^Centre of Biomedicine, Manchester Metropolitan University, Manchester, United Kingdom; ^3^Faculty of Biology, Medicine and Health, University of Manchester, Manchester, United Kingdom; ^4^Fortius Clinic, London, United Kingdom

**Keywords:** totally extraperitoneal repair, inguinal hernia, chronic groin pain, Fibrin sealant, atraumatic mesh fixation

## Abstract

**Introduction:** This study looks at the outcome of 352 patients that underwent the “Manchester groin repair” in the period from 2007 to 2016. The effect of laterality on chronic groin pain and the reduction of pain scores post-surgery are evaluated as well as the rate of hernia recurrence for the inguinal hernia repairs.

**Methods:** The “Manchester groin repair” is a modification of a laparoscopic totally extra-peritoneal approach with fibrin sealant mesh fixation. Data were collected prospectively. In addition to demographic data and the European Hernia Society classification grading of each hernia, pain scores were assessed prior to surgery and at 4–6 weeks post-operatively using a ten-point visual analog pain scale. Data were collected on a bespoke database and differences between time-points analyzed by non-parametric Wilcoxon signed rank tests with Kruskal-Wallis rank sum test for three-group comparisons. Significance was at the *P* < 0.05 level. The study was undertaken as an institutional audit.

**Results:** Three hundred and fifty two patients underwent TEP repair as per the “Manchester Groin Repair” modification during the period of interest with a median follow-up period of 109.5 (IQR 57.0–318.5) weeks. Of these 274 (77.8%) were for the repair of true hernias and 78 (22.2%) were for inguinal disruptions.

All inguinal hernia repairs patients were evaluated (254 m, 20 f); median [interquartile range] age 50 (39–65) years. There were 75 right inguinal hernias (27.4%), 39 Left inguinal hernias (14.2%), and 160 bilateral inguinal hernias (58.4%), giving a total of 434 hernia repairs. During follow-up there were 6 recurrences (1.4%).Of the 274 patients evaluated, 145 (52.9%) had both pre and post-operative pain scores available. Median pre-operative pain score was 5 [IQR 4–7]. Median post-operative pain score was 1 [IQR 1–2]. This difference was significant (*P* < 0.001). Pre-operative pain scores were higher for those with a bilateral hernia (median 6 vs. 5 and 4, respectively; *P* = 0.005), but there was no difference in post-operative scores (*P* = 0.347). One patient (0.3%) presented with chronic groin pain (pain after 3 months).

**Conclusion:** This study demonstrates that the “Manchester groin repair” provides an excellent repair with a low rate of recurrence and low incidence of chronic pain. Longer-term evaluation and larger patient series will add to the understanding of the role of this procedure in groin hernia repair.

## Introduction

The success of Inguinal Hernia repair is measured by the combination of a low recurrence rate together with a low incidence of post-repair chronic pain. Recently, with heightened media interest (1), there has been a focus on chronic post-surgery inguinal pain (CPIP) and its relationship to the use of mesh prostheses. Does this mean that the use of mesh could become a potential source for litigation? It has lead some to predict a re-emergence of tissue-only repair techniques (2). Despite this current criticism it is well recognized and accepted that reduced recurrence rates are historically and contemporaneously attributable to the widespread use of tension-free mesh repairs (3–5).

A consensus statement on CPIP in 2007 established its definition and alluded to numerous possible aetiologies of the pain that include the mesh itself and other materials used in traumatic mesh-fixation (6). Avoidance of CPIP is a primary goal for all inguinal hernia repairs and recommendations for achieving this include; (1) all three nerves are preserved and protected (avoidance of inadvertent nerve injury), (2) a minimal access technique is employed where available, (3) use of atraumatic mesh-fixation methods, and (4) use of a “lightweight” and flat mesh prosthesis (6–8). The quoted incidences of clinically significant CPIP is 10–12% with debilitating chronic pain affecting normal daily or work related activities at a very low rate of 0.5–6% (8, 9).

Laparoscopic or minimal access repair, in addition to a reduction in CPIP, have been shown to have an earlier return to normal activity and less immediate postoperative pain (8) (10&^12^). These benefits are further facilitated by the ongoing advancements in mesh technology.

The totally extra-peritoneal (TEP) repair is one minimal access technique that has been shown to provide a reduction in pain (CPIP) when performed with no method of mesh fixation (13–15) and when mesh is fixated with Fibrin Sealant (FS) (8, 16, 17). The use of FS incurs further benefits over a reduction in CPIP and combining the TEP repair with FS fixation, specifically ensuring that fixation in certain anatomical areas and with an increase in mesh size, was tested over a sustained period to try to demonstrate a reduced incidence of CPIP coupled with a low rate for hernia recurrence.

The Manchester groin repair has therefore been fashioned over the last 10 years in response to the rapidly changing landscape of inguinal hernia surgery, from open to minimal access, and as a product of the first-hand experience of a high-volume operator. To the author's knowledge, this is the first report of this TEP repair modification. It includes detailed explanation of the rationale for the technical elements described such as the novel approach to the localization of mesh-fixation using FS.

## Methods

This study relies on the use of contemporaneous data collected on a secure NHS trust database on patients who presented with uncomplicated primary and recurrent inguinal hernia or inguinal disruption. Careful follow up, including serial visual analog pain scores and assessment for recurrence, were documented. A qualified statistician undertook all subsequent analyses.

### Study design

A retrospective audit of patient and outcome data for patients undergoing inguinal hernia repair by the “Manchester Groin Repair” (see Table 1) using a prospectively upheld database at Manchester University NHS Foundation Trust.

**Table 1:** Table of comparison; Elements of Manchester Groin Repair c.f. Conventional TEP repair.

	**Manchester Groin Repair**	**Conventional TEP repair**
Inspection	Internal ring and obturator fascia	Internal ring mandatory only
Cooper's ligament	Dissection to 3 cm below Ilio-pectineal ligament	Dissection to ligament only or just below
Mesh type	“Lightweight” mesh	Surgeon's preference
Mesh size	15 × 12 cm mesh	15 × 10 cm mesh
Mesh fixation	Tissue glue; pubic tubercle, inferior edge, superior lateral edge, and obturator fascia	Tack fixation and no fixation described; inferior fixation not considered essential
Bilateral repair	Mesh crosses the mid line	Midline cover not always required

### Study period

Patients are included that underwent the “Manchester Groin Repair” from March 2007 to March 2017 (10 years).

### Patient selection

All patients aged 18 years and above were included in the study. Data was collected that includes: patient demographics, hernia type and size [as classified using the European Hernia Society (EHS) classification (18)], operating time, length of post-operative stay, perioperative morbidity, and episodes of recurrence.

A subgroup of patients was issued with approved pre- and post-operative quality of life questionnaires as described in a previous publication (19). Visual analog pain scores (VAS) depicting facial expressions with a scale of 0–10 were used with 0–1 being equivalent to very little or no pain. The pain chart, also with the added painful facial expressions, helped increase the understanding of the chart by patients.

Incomplete and unanswered questionnaires were followed up by a phone call for missing data. Patients were given a follow up appointment at 6 week post-surgery; non-attendees were followed up with a phone call.

### Operative technique

All hernia repairs were undertaken using a general anesthetic with the patient in a supine position. An 11 mm Dual balloon set (Pajunk® GmbH Geisingen, Germany) was introduced using a small incision just lateral to the umbilicus in the sub-rectus space and inflated under vision to create the extra-peritoneal space. Two 5 mm ports were sited under vision in a vertical line below the umbilicus and at least 3–5 cm above the symphysis pubis. Once the hernia was adequately reduced, a 15 × 12 cm polyester porous mesh Parietex^TM^ mesh (Medtronic TCM1515, Fridley, Minnesota, USA) cut to size was placed ensuring at least a 3 cm cover below Cooper's (ilio-pectineal) ligament medially and to the anterior superior iliac spine laterally. The mesh was fixated using 2 ml and 4 ml of fibrin sealant for unilateral and bilateral herniae, respectively (Tisseel™, Baxter, Newbury, UK). The sealant is ready to use and defrosted at the start of the operation and connected to the Duplospray MIS applicator® (Baxter, Vienna, Austria). The sealant was applied as per recommended practice issued by the manufacturer. Fibrin sealant was applied to ensure that the mesh was fixated over Cooper's ligament, the far lateral edge, along the inferio-lateral, and inferior-medial borders, obturator fascia as well as over the “triangle of doom' and pain”. The pneumoperitoneum was set at 12 mmHg with a flow rate of 20 l/min. Patients were discharged after review on the same day as surgery.

### Statistical analysis

Continuous data are presented as median range. A statistical analysis was undertaken by nonparametric test using the Mann-Whitney *U* test for two group comparisons, and the Kruskal-Wallis with a *post-hoc* Kruskal Nemenyi test for three groups, accepting a significance level at *P* < 0.05.

All statistical analyses were undertaken in R v3.0.2 (http://www.R-project.org/).

### Study approval

The study was registered as an approved audit with the then Central Manchester Foundation NHS Trust, now Manchester University NHS Foundation Trust. Ethics committee approval was sought and not required.

## Results

Three hundred and fifty two patients underwent TEP repair as per the “Manchester Groin Repair” modification during the period of interest with a median follow-up period of 109.5 (IQR 57.0–318.5) weeks. Of these 274 (77.8%) were hernia repairs, with 160 patients undergoing bilateral hernia repair simultaneously, giving an overall total of 434 hernia repairs. There were 35/434 repairs for recurrent hernia (8%). The remaining patients (78) underwent surgery for inguinal disruption (Table 2).

**Table 2:** Table showing number and percentage of patients as per indication for surgery.

**Hernia/disruption**	**Side**	**Number of patients**	**Number of groins operated**	**Percentage of total patients (*n* = 352)**
Hernia	Right inguinal	75	75	21.3
	Left inguinal	39	39	11.1
	Bilateral inguinal	160	320	45.5
	Total	274	434	77.8
Disruption	Right inguinal	5	5	1.4
	Left inguinal	8	8	2.3
	Bilateral inguinal	65	130	18.5
	Total	78	143	22.2
Overall total		352	577

We experienced six episodes of recurrence (1.4%). All recurrences were detected after 3 months on clinical evaluation at the time of follow up. However, on subsequent corrective surgery, no true hernia was evident in any patient other than a lipoma of the cord. All recurrences were re-operated on by the open technique and required a non-absorbable suture repair of the internal ring only.

One patient (0.3%) presented with chronic post-surgery inguinal pain (CPIP), defined as pain after 3 months (6), with a visual analog pain score of 6.

For all subsequent analyses we exclude the disruptions to leave 274 patients.

**Figure 1:**
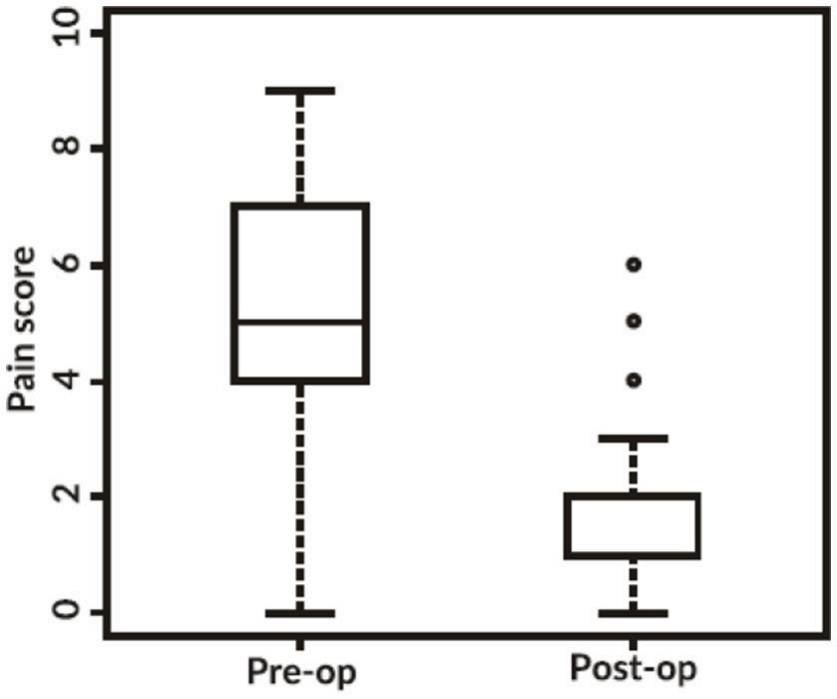
Box plot demonstrating the pre-op and post-op pain scores reported (*p* < 0.001).

Of the 274 patients pain scores are complete for 145 individuals. The following boxplot (Figure 1) demonstrates that before surgery the pain scores were widely spread, almost across the whole range in a slightly skewed distribution, (median 5, IQR 4–7), while after surgery there is a distinctive drop with 64.8% of individuals scoring 1 or less and only a few outliers (eight) remain over 4 (median 1, IQR 1–2). Testing this change with a Wilcoxon signed rank test gives a significant *p*-value < 0.001, therefore we reject the hypothesis that the two pain scores come from similar distributions. Hence, the overall difference between time-points is statistically significant, showing a reduction in pain after surgery.

## Unilateral vs. bilateral hernias

A comparison was made between unilateral and bilateral hernias for the 145 with pain scores. Those with a bilateral hernia appeared to have a larger degree of pain pre surgery (median [IQR] 6 [4.00–8.00] vs. 5 [2.75–6.00]) which falls to a similar median level [1, [1,2] and 1, [0–2], respectively] post. Testing the change scores to see whether they come from a similar distribution, the *p*-value of 0.0159 suggests we should reject the null hypothesis that the changes in the two groups are from similar distributions. In conclusion the difference in pain between uni and bilateral hernias lies pre-surgery, with a greater change seen in bilateral hernias. Whether the hernia was uni or bilateral did not affect the pain scores post operatively.

## Left, right vs. bilateral hernias

Those with a bilateral hernia appear to have a larger degree of pain pre surgery, followed by the left side (median [IQR] 6 [4.0–8.0] vs. 5 [4.5–6.0] vs. 4 [2.0–6.0]), all of which fall to a median level of 1 [IQR [1,2], [0–2], and [1,2], respectively] post-surgery. Testing the change scores using a Kruskal Wallis test to see whether they come from a similar distribution, the *p*-value 0.040 suggests we should reject the null hypothesis that the changes in the three groups are from similar distributions. The *post-hoc* test suggests that the change in pain scores is significantly different for those with bilateral vs. right hernias (*p* = 0.035).

## Operation group

Of the patients 144 have complete pain scores and a defined operation group. Although the different groups have different levels of pain prior to surgery, afterwards all seem to decrease to similar small levels. A Kruskal-Wallis test gives a *p*-value of 0.329 suggesting no difference between the change in pain in the operation groups (Table 3).

**Table 3:** Operation group as per the EHS classification showed no difference in the pre and post-operative pain scores (*P* = 0.329).

**Operation group**	***N***	**Pre-surgery**		**Post-surgery**		**Change**	
		**Median**	**IQR**	**Median**	**IQR**	**Median**	**IQR**
L1	29	5	4.00–7.00	1	1.00–2.00	−4	−5.00 to −2.00
L2	57	6	4.00–8.00	1	1.00–2.00	−5	−6.00 to −2.00
L3	15	6	4.00–8.00	1	1.00–1.50	−5	−7.00 to −3.00
Ml	16	5	2.75–6.00	1	0.00–2.00	−3	−5.25 to −1.75
M2	20	4	4.00–6.00	1	1.00–2.25	−3	−4.00 to −2.00
M3	7	3	2.00–5.00	1	0.00–1.00	−2	−4.50 to −1.50

In linear regression analyses there was no effect shown in terms of laterality of hernia and degree of post-operative pain.

## Morbidity

Only 32 patients suffered from a complication giving an overall morbidity of 9%. The commonest complication noted was a seroma in 12 patients (3.4%) but only 1 of them required an intervention with needle aspiration. Other complications included hydrocele of the cord, haematoma formation and testicular discomfort. There were no re-operations within 3 months and no early recurrences as well as any reported deaths or re-hospitalization.

## Discussion

Hernia surgery remains a technique driven surgical entity. What constitutes a good repair remains a contentious question and in consequence any particular repair can be recommended by a surgeon with a high volume practice depicting evidence of good outcomes (8, 20). Recently published groin hernia guidelines promote a safe and evidence guided practice for hernia surgery (8, 21). In these guidelines, recommendations are made based on mesh type, fixation technique and the method of repair (8, 21). This includes a recommendation for the use of atraumatic fixation methods, such as the use of FS and other “glues”, in reducing the incidence of CPIP (level 1b) (8). Mesh fixation is mandated for large medial hernias, M3 as per the EHS classification of groin hernias (grade of recommendation A) (8, 21). In the case of other smaller hernias, no-fixation has been deemed both safe and effective (8).

Earlier publications evaluated surgical management options for patients suffering with chronic pain following IHR where various methods of treatment were examined; mesh removal, selective neurectomy, removal of surgical clips as well as the use of diagnostic, and therapeutic analgesics and anesthetic agents. The overall level of evidence included in this review was not of a robust enough standing by being based on largely subjective data and evidence. Therefore, despite the reports of favorable outcomes in individual cases, no clear conclusions can be drawn on what exactly causes CPIP, it is likely that a multitude of factors are responsible (22). Despite this, the rational-minded surgeon can be led to believe that increasing traumatic injury to an area of tissue, particularly containing nerves, poses an increased risk of neuropathic symptoms and may subsequently contribute to CPIP (8). One clear benefit of tissue glue is the ability to apply it to these “high risk” areas and achieve mesh anchorage without the risk of trauma to the underlying structures. Three reviews comparing permanent versus non-permanent fixation in TEP repair have depicted advantages of glue-fixation in reducing the incidence of chronic pain (16, 17, 23).

The use of FS, a composite of fibrinogen and thrombin preparations, is now a well-established and recognized method for mesh-fixation. For TEP repair, mechanical integrity was first shown in animal experiments (24) and also a reduction in the need for post-operative analgesia was demonstrated in a randomized controlled trial setting (25) as was a quicker return to physical and social activities (26). A systematic review of the use of FS compared to staples in TEP repair concluded that there was no superiority in terms of hernia recurrence and that FS fixation might be the preferable technique based on its associated decreased incidence of CPIP (17). Recent retrospective analyses of case series have shown favorable patient centered outcomes with low incidences of CPIP and recurrence (19, 27).

The “Manchester Groin Repair” relies on the use of FS to achieve fixation in key areas, namely the inferiolateral and inferiomedial edges as well as at least 3 cm below the ilio-pectineal ligament on the obturator fascia, using a slightly larger (15 × 12 cm) mesh size than that which is commonly used (15 × 10 cm) for laparoscopic repair.

In the author's experience, currently very few open and laparoscopic surgeons opt for a no-fixation method of mesh placement in day-to-day practice. This move toward no-fixation may be too much of a “jump”. Given the aforementioned recommendation that there is still a need to fixate mesh prostheses in some instances, namely a large direct hernia (M3 and above) (8), it is likely that it will take some time and considerable evidence for no-fixation to be widely adopted in practice. With the possibility that self-gripping mesh technology may play a role in advancing this movement (28).

Recent international guidelines have reviewed 23 RCTs relating to mesh material and clinical outcomes and subsequently state that the evidence supports the contention that mesh characteristics influence clinical outcomes (8). Key characteristics of a prosthesis that should be commonplace in the rationale for groin hernia repair include; mesh size, mesh material, mesh construct and the size of the pores within the mesh. Contemporary advice from recent international guidelines is states that the use of the weight of an implant as a singular parameter for mesh classification is no longer acceptable (8). This case series reporting the use of the Manchester groin repair had already begun ahead of the publication of the aforementioned guidelines. The choice of a “Lightweight Mesh” refers to a mesh construct with large-pore size and reduced weight. These factors make a difference with the larger pore size significantly improving surrounding soft tissue integration (29, 30) and reducing fibrotic bridging between filaments and the subsequent risk of excessive mesh contraction (31). Randomized studies have shown advantages in the use of “lightweight mesh” compared to “heavyweight mesh” in TEP repair with reduced post-operative pain and foreign body sensation with no real effect on sexual function (32–34).

Any mesh prosthesis used is required to obtain adequate coverage of the hernia defect and so reduce the risk of recurrence. Study of the TEP repair with a 15 × 7.6 cm mesh prosthesis resulted in unacceptably high levels of hernia recurrence and it was therefore recommended that a 15 × 10 cm mesh prosthesis be used as a minimum (35, 36). In this modification of the TEP repair, increased dissection allows for the easy placement of a 15 × 12 cm mesh prosthesis. The author alludes to the fact that with the increased coverage and subsequent mesh associated strengthening of a wider area, in addition to the added effect of the enhanced collagen deposition as a result of the use of FS, will have contributed to the lower level of hernia recurrence and possibly too the incidence of CPIP.

This study, albeit with not large numbers, provides more evidence for atraumatic mesh-fixation with FS. In addition, it outlines the role of selective application of FS at key areas within the groin. It includes the recommendation of a relative increase in dissection to be undertaken; at least 3 cm below the ilio-pectineal “Cooper's” ligament. This creates a space for mesh-fixation to the obturator fascia. The results demonstrate that this technique has the potential to be highly efficacious. A reported low rate of recurrence (1.4%) and very low incidence of CPIP (0.3%) especially with a median follow up of 110 weeks, the author feels that these technical adaptations are in keeping with the steps that hernia surgeons will be willing to undertake in a transition toward atraumatic mesh-fixation and no-fixation during inguinal hernia repair.

## Conclusion

This case series provides evidence and rationale of a safe and efficacious modification of the established TEP repair. This information supports that a surgeon establishing laparoscopic hernia practice will consider the use of a lightweight, wide-pore mesh of a good size (15 by 12 cm) and achieve mesh-fixation with an atraumatic method e.g., Fibrin Sealant (FS). This combination assures a relative low incidence of post-herniorrhaphy pain, both acute and chronic, and a low incidence of hernia recurrence.

## Limitations

Data from a high volume hernia surgeon; is the high volume nature of practice the real benefactor to the favorable results seen here or is it the technical alterations (most likely a combination of the two).

The numbers are small but the follow up spans over a long period, but although not all patients were able to provide VAS pain scores.

## Ethics statement

Ethics approval was sought and advised that this retrospective data analysis is exempt from Ethics and this has been defined in the methodology. The study was registered as an audit with Manchester University Foundation NHS Trust.

## Author contributions

JP: Writing of manuscript and data analysis. MO, MZ, PS, AKS, and SJ: Data analysis, editing of manuscript. AJS: Operating surgeon, data analysis, writing and editing of manuscript.

### Conflict of interest statement

The authors declare that the research was conducted in the absence of any commercial or financial relationships that could be construed as a potential conflict of interest.
